# Metabolism of Daidzein and Genistein by Gut Bacteria of the Class *Coriobacteriia*

**DOI:** 10.3390/foods10112741

**Published:** 2021-11-09

**Authors:** Sebastian Tobias Soukup, Dominic Alexander Stoll, Nicolas Danylec, Alena Schoepf, Sabine Emma Kulling, Melanie Huch

**Affiliations:** Department of Safety and Quality of Fruit and Vegetables, Federal Research Institute of Nutrition and Food, Max Rubner-Institut, Haid-und-Neu-Straße 9, 76131 Karlsruhe, Germany; sebastian.soukup@mri.bund.de (S.T.S.); dominic.stoll@mri.bund.de (D.A.S.); nicolas.danylec@hotmail.de (N.D.); alena.schoepf@outlook.de (A.S.); sabine.kulling@mri.bund.de (S.E.K.)

**Keywords:** *Eggerthellaceae*, *Coriobacteriaceae*, anaerobic, isoflavones, daidzein, genistein, equol, microbial, metabolism

## Abstract

The intake of isoflavones is presumed to be associated with health benefits in humans, but also potential adverse effects of isoflavones are controversially discussed. Isoflavones can be metabolized by gut bacteria leading to modulation of the bioactivity, such as estrogenic effects. Especially bacterial strains of the *Eggerthellaceae,* a well-known bacterial family of the human gut microbiota, are able to convert the isoflavone daidzein into equol. In addition, metabolization of genistein is also described for strains of the *Eggerthellaceae*. The aim of this study was to identify and investigate gut bacterial strains of the family *Eggerthellaceae* as well as the narrowly related family *Coriobacteriaceae* which are able to metabolize daidzein and genistein. This study provides a comprehensive, polyphasic approach comprising in silico analysis of the equol gene cluster, detection of genes associated with the daidzein, and genistein metabolism via PCR and fermentation of these isoflavones. The in silico search for protein sequences that are associated with daidzein metabolism identified sequences with high similarity values in already well-known equol-producing strains. Furthermore, protein sequences that are presumed to be associated with daidzein and genistein metabolism were detected in the two type strains ‘*Hugonella massiliensis*’ and *Senegalimassilia faecalis* which were not yet described to metabolize these isoflavones. An alignment of these protein sequences showed that the equol gene cluster is highly conserved. In addition, PCR amplification supported the presence of genes associated with daidzein and genistein metabolism. Furthermore, the metabolism of daidzein and genistein was investigated in fermentations of pure bacterial cultures under strictly anaerobic conditions and proofed the metabolism of daidzein and genistein by the strains ‘*Hugonella massiliensis*’ DSM 101782^T^ and *Senegalimassilia faecalis* KGMB04484^T^.

## 1. Introduction

**Figure 1:**
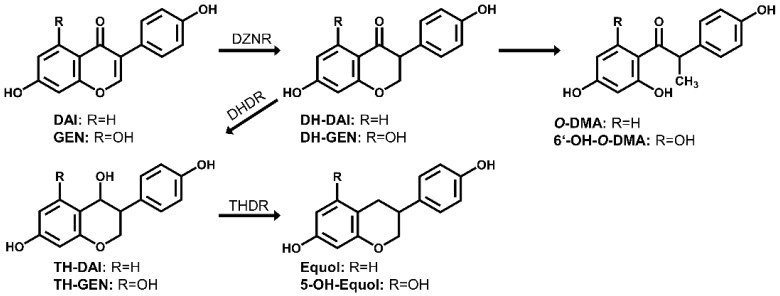
Metabolism of daidzein and genistein by human gut bacteria. Modified based on [6,7]. Daidzein (DAI); genistein (GEN); dihydrodaidzein (DH-DAI); dihydrogenistein (DH-GEN), tetrahydrodaidzein (TH-DAI); tetrahydrogenistein (TH-GEN); 5-hydroxyl-equol (5-OH-equol); *O*-desmethylangolensin (*O*-DMA); 6′-hydroxy-*O*-desmethylangolensin (6′-OH-*O*-DMA); daidzein reductase (DZNR), dihydrodaidzein reductase (DHDR); tetrahydrodaidzein reductase (THDR).

Daidzein and genistein, two isoflavones with a very similar molecular structure, are well-known to be present in soy and soy-based food [1]. Daidzein and genistein belong to the phytoestrogens as they are “biologically active phenolic compounds derived from plants” with “structures similar to the principal mammalian estrogen” [2]. Several health benefits associated with the consumption of isoflavones are discussed, such as the alleviation of menopausal symptoms, prevention of osteoporosis, and improvement of cancer prognosis [3]. Besides beneficial effects, potential adverse effects of isoflavone intake are also controversially discussed [4]. These effects are often associated with their estrogenic activity. For isoflavones the endogenous transformation in humans is well known and they can be metabolized by endogenous phase I and phase II enzymes [4]. Besides that, isoflavones can be metabolized by gut bacteria leading to modulation of their bioactivity. High inter-individual variations in this microbial metabolism are observed in humans. For example, only about one-third of the human population can convert daidzein to equol, an important metabolite showing the highest affinity to estrogen receptors (ER) among all known isoflavones [4]. Especially strains of the family *Coriobacteriaceae* are able to convert food polyphenols like daidzein [5]. However, there is still limited knowledge of bacteria that are involved in this conversion [6]. In the intestine, daidzein can be reduced by bacterial metabolism either to equol via dihydrodaidzein (DH-daidzein) and tetrahydrodaidzein (TH-daidzein) and/or to *O*-desmethylangolensin (*O*-DMA) also via DH-daidzein [6,7] (Figure 1). Similar to the metabolization of daidzein, genistein can be converted by bacterial action to 6′-hydroxy-*O*-desmethylangolensin (6′-OH-*O*-DMA) [4]. The formation of 5-hydroxyl-equol (5-OH-equol) from genistein is described for the type strains of *Adlercreutzia mucosicola, Slackia equolifaciens* and *Slackia isoflavoniconvertens* [8,9,10]. However, to the best of our knowledge, 5-OH-equol has not yet been detected in human biofluids, so evidence for its formation in vivo is still lacking. It is well-known that not every bacterial strain that is able to metabolize e.g., daidzein can conduct each single metabolization step from daidzein to equol, which is probably the end product of the metabolic transformation. Some strains only convert daidzein to intermediate metabolites or are able to further metabolize intermediate metabolites [6].

Since especially strains of the *Coriobacteriaceae* and *Eggerthellaceae* are associated with the metabolization of daidzein and genistein, it has to be noted that taxonomic changes within the parent class *Coriobacteriia* occurred [11,12]. The following *Eggerthellaceae* strains are described to metabolize daidzein and/or genistein: *Adlercreutzia equolifaciens* subsp. *celatus* DSM 18785^T^ [13], *A*. *equolifaciens* subsp. *equolifaciens* DSM 19450^T^ [14], *Adlercreutzia mucosicola* DSM 19490^T^ [15], *Eggerthella* sp. YY7918 [16], *Slackia equolifaciens* DSM 24851^T^ [17], *Slackia isoflavoniconvertens* DSM 22006^T^ [8], *Slackia* sp. AUH-JLC159 [18], and *Slackia* sp. NATTS [19]. The metabolization of daidzein to O-DMA is described for *Slackia exigua* DSM 15923^T^ [8,14]. The enzymes that are involved in daidzein and genistein metabolism were identified and characterized in five bacterial strains, i.e., *Lactococcus garvieae* 20–92 [20,21,22], *S. isoflavoniconvertens* DSM 22006^T^ [23], *Slackia* sp. NATTS [24], *A. equolifaciens* DSM 19450^T^ [25] and *Eggerthella* sp. YY7918 [16]. The following three genes are described to be necessary for equol production: I) daidzein reductase (DZNR), II) dihydrodaidzein reductase (DHDR), and III) tetrahydrodaidzein reductase (THDR). A fourth enzyme (dihydrodaidzein racemase, DDRC) is described to increase equol production [22,23]. The corresponding genes that are associated with the metabolism of daidzein and genistein are located in one cluster [6,25], named equol gene cluster throughout the manuscript. DH-daidzein and equol occur in two enantiomeric forms; *S*-equol is the exclusive enantiomeric form that is produced by human intestinal bacteria [22,26]. DDRC may catalyze the conversion of *R*- into *S*-DH-daidzein and may explain the exclusive *S*-equol production [22,26].

Due to the similar molecule structure, it is presumed that daidzein converting enzymes also metabolize genistein which is already confirmed for the daidzein reductase of *S. isoflavoniconvertens* DSM 22006^T^ [23]. However, strain *Eggerthella* sp. YY7918 converts daidzein, but not genistein [27]. The metabolization of genistein seems to be less investigated than the metabolization of daidzein. However, genistein metabolization is described, for e.g., *A. mucosicola* DSM 19490^T^ [15] and *S. isoflavoniconvertens* DSM 22006^T^ [8].

The aim of this study was to investigate the metabolism of daidzein and genistein in gut-related bacterial strains of the families *Eggerthellaceae* and *Coriobacteriaceae*. Firstly, an in silico approach detecting genes of the equol gene cluster in recently annotated draft genome sequences of strains of these two families was conducted. In addition, a comprehensive PCR approach of 29 strains was performed to search for the presence of genes of the equol gene cluster. Conclusively, the metabolism of daidzein and genistein was investigated by fermentation of pure cultures of these 29 *Eggerthellaceae* and *Coriobacteriaceae* strains under strictly anaerobic conditions.

## 2. Materials and Methods

**Chemicals**. The purity of all analytes was determined by LC-DAD (220–600 nm). Daidzein (purity of 99.6%), (*R*, *S*)-equol (99.9%), and genistein (99.7%) were purchased from LC Laboratories (Woburn, MA, USA). Dihydrodaidzein (99.7%) and *O*-DMA (95.9%) were purchased from Toronto Research Chemicals (Toronto, ON, Canada) and Plantech UK (Reading, UK), respectively. DH-genistein (97.8%) and 6′-OH-*O*-DMA (97.0%) were purchased from APIN Chemicals (Abingdon, UK). All other chemicals used were of analytical grade. Deionized water was taken from the in-house ultrapure water system LaboStar (Siemens) with a conductivity of 0.055 μS/cm.

**Strains and culture conditions**. For comparative analysis, bacterial strains were obtained from the German Collection of Microorganisms and Cell Cultures GmbH (DSMZ), the Japanese Culture Collection (JCM), the Collection de Souches de l’Unité des Rickettsies, Unités des Rickettsies, France (CSUR), the Korean Collection for Type Cultures (Korea Research Institute of Bioscience and Biotechnology) (KGMB) and the own laboratory collection (MRI-F and ResAG): *Adlercreutzia equolifaciens* subsp. *equolifaciens* DSM 19450^T^, *Adlercreutzia equolifaciens* subsp. *celatus* DSM 18785^T^*, Adlercreutzia rubneri* ResAG-91^T^, *Adlercreutzia caecimuris* DSM 21839^T^*, Adlercreutzia mucosicola* DSM 19490^T^*, Adlercreutzia muris* DSM 29508^T^*, Cryptobacterium curtum* DSM 15641^T^, *Denitrobacterium detoxificans* DSM 21843^T^*, Eggerthella lenta* DSM 2243^T^*,* DSM 15644, *Eggerthella sinensis* DSM 16107^T^, *‘Eggerthella timonensis’* CSUR P3135^T^, *Ellagibacter isourolithinifaciens* DSM 104140^T^*, Enteroscipio rubneri* ResAG-96^T^*,* ‘*Gordonibacter massiliensis’* CSUR P2775^T^, *Gordonibacter pamelaeae* DSM 19378^T^, *Gordonibacter urolithinfaciens* DSM 27213^T^, JCM 16058, *‘Hugonella massiliensis’* DSM 101782^T^, *Paraeggerthella hongkongensis* DSM 16106^T^, *Parvibacter caecicola* DSM 22242^T^, *Rubneribacter badeniensis* ResAG-85^T^*, Slackia exigua* DSM 15923^T^*, Slackia equolifaciens* DSM 24851^T^*, Slackia faecicanis* DSM 17537^T^*, Slackia heliotrinireducens* DSM 20476^T^, *Slackia isoflavoniconvertens* DSM 22006^T^, *Slackia piriformis* DSM 22477^T^*, Senegalimassilia anaerobia* DSM 25959^T^ and *Senegalimassilia faecalis* KGMB04484^T^. Cultivation of all strains was performed at 37 °C under strictly anaerobic conditions either in Hungate tubes flushed with N_2_/CO_2_ (80/20) or in an A45 anaerobic workstation (Don Whitley Scientific) under atmospheric conditions of N_2_/CO_2_/H_2_ (80/10/10). All strains were cultured for 48–72 h in modified BHI (5 g L^−1^ yeast extract, 0.05 g L^−1^ l-cysteine monohydrochloride, 1 mg L^−1^ resazurin sodium salt, 2.5 mg L^−1^ haem solution, 2 μg mL^−1^ vitamin K1 solution).

**In silico identification of the equol gene cluster**. The annotated draft genome sequence of *S. isoflavoniconvertens* DSM 22006^T^ (QIBZ00000000) [28] was used as a reference genome. The equol gene cluster was localized by using the protein sequences (DZNR: AFV15453; DHDR: AFV15451; THDR: AFV15450; DDRC: AFV15447) of the respective genes. These protein sequences were used to perform a protein BLASTp search. Clustering of the protein sequences was performed using BioNumerics (version 8.0, Applied Maths). Genome segments and annotations of the respective strains were visualized, and analyzed by the use of the CLC Sequence Viewer software (version 8, Qiagen).

**DNA Isolation, PCR, agarose gel electrophoresis, and clustering**. Bacterial cells (10 mL) were harvested by centrifugation (10 min, 4 °C, 6540× *g*) and genomic DNA was isolated using the blood and tissue kit (Qiagen) for Gram-positive bacteria according to the manufacturer’s instructions. DNA was quantified with the double-stranded DNA (dsDNA) high-sensitivity (HS) assay kit on a Qubit version 2.0 fluorometer (Thermo Fischer Scientific) and was adjusted to a concentration of 10 ng µL^−1^. Gene-specific primers for *dzr* (dzr.qPCR-F: 5′-GAA GCT TGA TAT GGA CGA CT-3′; dzr.qPCR-R: 5′-GGA ATA TGC ACC TGT TCC T-3′), *ddr* (ddr.qPCR-F: 5′-CTC GAY CTS GTS TAC AAC GT-3′; ddr.qPCR-R: 5′-GAR TTG CAG CGR ATK CCG AA-3′) and *tdr* (tdr.qPCR-F: 5′-RTY AAC GGC RAY ATG CAG GT-3′; tdr.qPCR-R: 5′-GGM AYY TCC ATG TTG TAG GA-3′) developed by [29] were used. Each PCR reaction consisted of 5 µL of DNA template, 1.25 µL of each of the respective primer (10 pmol/µL; synthesized by biomers.net), 12.5 µL of ALLin Hot Start Taq Mastermix 2× (HighQu), and 5 µL PCR Water (HighQu). PCR was performed in a peqSTAR 96 Universal Thermocycler (PeqLab) with the following conditions: Initial degradation at 94 °C for 4 min, followed by 30 cycles (94 °C 45 s, 60 °C 45 s, 72 °C 30 s) and a final elongation at 72 °C for 6 min. Genomic DNA of *S*. *isoflavoniconvertens* DSM 22006^T^ was used as positive control. PCR products were subjected to electrophoresis on a 3% agarose gel containing ethidium bromide (120 min, 100 V). PCR products were visualized using the gel documentation system Gel Doc XR+ (BioRad).

**Metabolization of daidzein and genistein by pure bacterial cultures**. The preparation of pure culture experiments was conducted in an anaerobic workstation under strictly anaerobe conditions of N_2_/CO_2_/H_2_ (80/10/10) at 37 °C. Bacterial pure strains were precultured in Hungate tubes flushed with N_2_/CO_2_ (80/20) at 37 °C. A volume of 100 µL of bacterial precultures was used to inoculate 10 mL modified BHI (5 g L^−1^ yeast extract, 0.05 g L^−1^ l-cysteine monohydrochloride, 1 mg L^−1^ resazurin sodium salt, 2.5 mg L^−1^ haem solution, 2 μg mL^−1^ vitamin K1 solution) in Hungate tubes. Fermentation was performed with 100 µL isoflavone (8 mM daidzein or 8 mM genistein in DMSO; final concentration 78.4 µM) in triplicates. The growth of strains was visually confirmed. In addition, two controls (without bacterial suspension and without isoflavone but 100 µL DMSO) were conducted for each trial. Samples (2 × 500 µL) were taken at 0, 24, 48, and 72 h and bacterial cells were removed by centrifugation (10 min, 4 °C, 15,000× *g*). The supernatant (2 × 400 µL) was stored at −80 °C until further analysis.

**LC-DAD and LC-MS analyses of fermentation samples**. Sample clean-up was performed according to [30] with some modifications. Samples were prepared on ice whenever this was possible. Briefly, supernatants from fermentations were thawed on ice and shortly vortexed. A sample volume of 400 µL was extracted three times with each 500 µL extraction solvent (ethyl acetate/isopropanol/1-butanol, 90/5/5, *v*/*v*/*v*). The combined organic layers were dried under a constant stream of nitrogen. Samples were dissolved in 20 µL DSMO followed by the addition of 180 µL dissolvent (0.1% aqueous formic acid/acetonitrile/methanol, 90/5/5, *v*/*v*/*v*). Afterwards, samples were vortexed (5 s) and centrifuged (5 min, 4 °C, 23,100*× g*). A volume of 180 µL of the supernatant was transferred to a vial and stored at 4 °C until further analysis. Samples were analyzed on a Prominence HPLC system (Shimadzu Europa GmbH, Duisburg, Germany) consisting of a controller (CBM-20A), degasser (DGU-20A3), two pumps (LC-20AD), a column oven (CTO-20AC), and an autosampler (SIL-20AC HT) coupled with an SPD-M20A diode array detector (DAD). Chromatography was carried out on a Cortecs C18 column (3.0 × 150 mm, 2.7 μm particle size; Waters GmbH, Eschborn, Germany) equipped with a pre-column (Cortecs C18, 2.1 × 5 mm, 2.7 μm particle size; Waters GmbH, Eschborn, Germany). Concerning the analysis of daidzein and its metabolites, 0.1% aqueous formic acid and methanol/acetonitrile (1/1, *v*/*v*) were used as eluents A and B, respectively. A flow rate of 0.7 mL/min was adjusted and the following gradient elution profile was used: 0.0–0.5 min isocratic with 25.5% B, 0.5–11.5 min from 25.5–42% B, 11.5–12.0 min from 42–99% B, 12.0–14.5 min isocratic with 99% B, 14.5–15.0 min from 99–25.5% B, and 15.0–20.0 min isocratic with initial conditions. Concerning the analysis of genistein and its metabolites, 0.1% aqueous formic acid and acetonitrile were used as eluents A and B, respectively. A flow rate of 0.7 mL/min was adjusted and the following gradient elution profile was used: 0.0–0.5 min isocratic with 23% B, 0.5–7.5 min from 23–30% B, 7.5–8.0 min from 30–99% B, 8.0–10.5 min isocratic with 99% B, 10.5–11 min from 99–23% B, and 11.0–16.0 min isocratic with initial conditions. For both methods, the column oven was set to 40 °C and the injection volume was 10 μL. The DAD recorded data from 200 to 600 nm with a sampling rate of 6.25 Hz, and a trace at 275 nm was used to monitor the analytes. The identity of each analyte was confirmed by the retention time and the UV spectrum. The system was controlled by the software LC solution 1.24 (Shimadzu Europa GmbH, Duisburg, Germany). The analysis method to quantify daidzein, DH-daidzein, (*R*, *S*)-equol and *O*-DMA as well as genistein, DH-genistein, and 6′-OH-*O*-DMA were validated and the parameters accuracy, precision, recovery, the limit of detection (LOD), limit of quantitation (LOQ), and linearity were determined. The results of the validation are given in Supplemental Data S13. For daidzein and genistein metabolization, *S. equolifaciens* DSM 24851^T^ [17] was used as a positive control. In addition, unknown metabolites of genistein were identified by accurate LC-MS (QToF) analysis: Samples were measured using a Triple TOF 5600 mass spectrometer (AB Sciex) linked to a 1290 Infinity LC system (Agilent). The LC-DAD-MS system was controlled by the software Analyst TF (version 1.8, AB Sciex, Darmstadt, Germany). Chromatography was performed as described above. Samples were measured both in the negative and in the positive mode. The DuoSpray source operated in electrospray ionization (ESI) mode using the following source parameters: Curtain gas 45 psi, ion spray voltage −4500 V and +5500 V, respectively, ion source gas-170 psi, ion source gas-260 psi, and ion source gas-2 temperature 650 °C. The declustering potential was adjusted to −100 V and +100 V, respectively. The MS full scans were recorded from *m*/*z* 100 to 1000 with an accumulation time of 100 ms and a collision energy voltage of −10 V and +10 V, respectively. The MS/MS spectra (product ion) were recorded from *m*/*z* 50 to 1000 in the high sensitivity mode with an accumulation time of 40 ms, a collision energy voltage of −35 V, and +35 V and a collision energy spread of 15 V. Nitrogen was used as collision gas. Data were analyzed using the software PeakView 2.2.0 and FormulaFinder 2.2.0 (AB Sciex, Darmstadt, Germany).

## 3. Results and Discussion

**In silico approach to identify the equol gene cluster.** The annotated draft genome sequence (NZ_QIBZ00000000.1) of the equol and 5-OH-equol-producing strain *S. isoflavoniconvertens* DSM 22006^T^ which was sequenced by our group previously [28] was used as a reference for the in silico approach. A comparison of the respective section on contig 17 (NZ_QIBZ01000017) of our annotated draft genome of *S*. *isoflavoniconvertens* DSM 22006^T^ and the sequence of the equol gene cluster (JQ358709) generated by Sanger sequencing [23] showed an identity of 100% of the nucleotide sequences.

**Figure 2:**
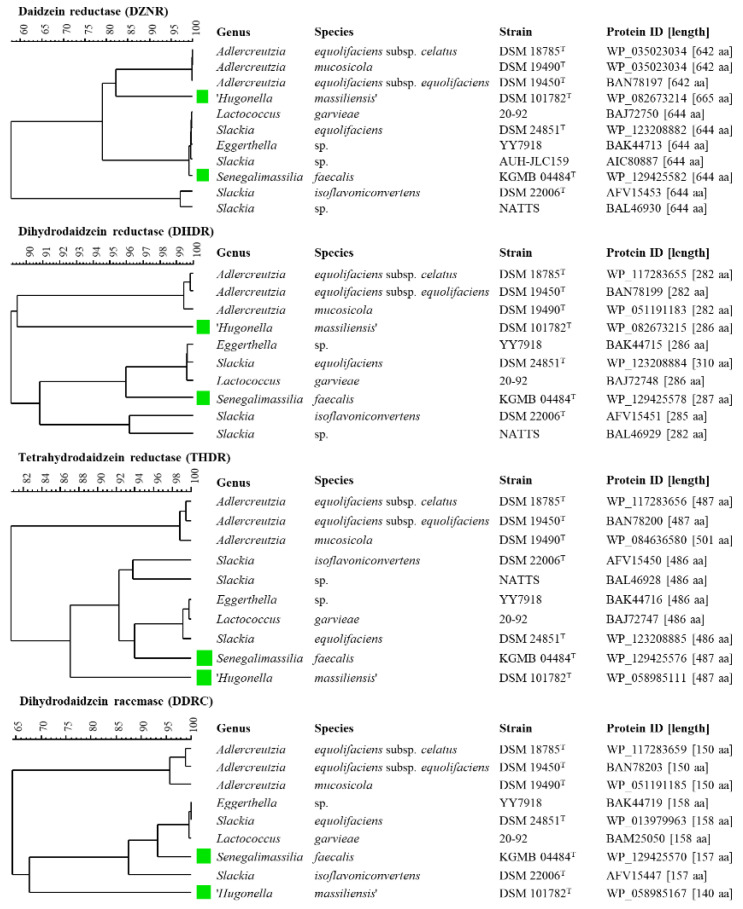
Cluster analyses of amino acid sequences of daidzein reductase (DZNR), dihydrodaidzein reductase (DHDR), tetrahydrodaidzein reductase (THDR), and dihydrodaidzein racemase (DDRC) of strains of the *Eggerthellaceae* and *Coriobacteriaceae*. Clustering was performed by multiple-alignment and UPGMA (unweighted pair group method with arithmetic mean) in BioNumerics 8.0. Green labeled strains are not yet described in the literature to be associated with daidzein or genistein metabolism, to the best of our knowledge.

The aim of this in silico study was to identify strains of the *Eggerthellaceae* and *Coriobacteriaceae* which harbor genes involved in daidzein and genistein metabolism. As a comparison of amino acids sequences rather than nucleotide sequences has tremendous advantages [31], the sequences of proteins of *S*. *isoflavoniconvertens* DSM 22006^T^ involved in daidzein metabolism, namely DZNR (AFV15453), DHDR (AFV15451), THDR (AFV15450), and putative DDRC (AFV15447), were used for BLASTp homology search. This search showed proteins with high similarity values in the following well-known equol-producing strains: *A*. *equolifaciens* subsp. *equolifaciens* DSM 19450^T^ [14], *A*. *equolifaciens* subsp. *celatus* DSM 18785^T^ [13,32], *A*. *mucosicola* DSM 19490^T^ [9], *S*. *equolifaciens* DSM 24851^T^ [17], *Lactococcus garvieae* 20–92 [33], *Slackia* sp. AUH-JLC159 [18], *Slackia* sp. NATTS [19], *Eggerthella* sp. YY7918 [34]. In addition to *S. isoflavoniconvertens* DSM 22006^T^, the occurrence of the equol gene cluster was already described for *A*. *equolifaciens* subsp. *equolifaciens* DSM 19450^T^ [35], *Lactococcus garvieae* 20–92 [33], *Slackia* sp. AUH-JLC159 [18], *Slackia* sp. NATTS [19], *Eggerthella* sp. YY7918 [34] and discussed in [6]. Furthermore, this BLASTp search showed protein sequences with high similarity to DZNR, DHDR, THDR, and putative DDRC in the type strains ‘*Hugonella massiliensis*’ DSM 101782^T^ [36] and *Senegalimassilia faecalis* KGMB04844^T^ [37]. This suggests that these strains might be capable of metabolizing daidzein and genistein. All identified protein sequences were compared by performing a multiple alignment followed by cluster analysis using the unweighted pair group method with arithmetic mean (UPGMA) (Figure 2). These comparisons showed lower similarity values within DZNR (≥57.8%) and DDRC (≥64.3%) than within DHDR (≥89.0%) and THDR (≥80.7%). Comparable findings of the similarities of DZNR, DDRC, DHDR, and THDR were described by [23].

**Figure 3:**
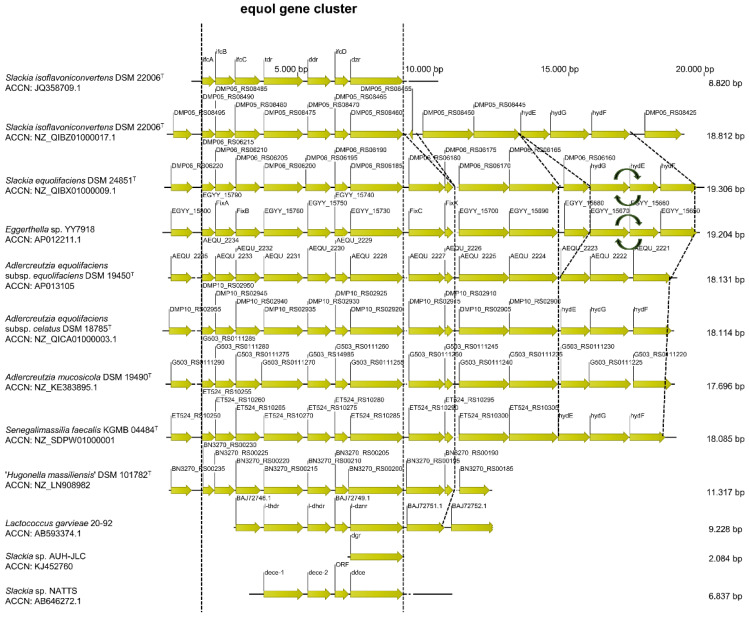
Schematic representation of the gene organization within the equol gene cluster (dihydrodaidzein racemase (DDRC), tetrahydrodaidzein reductase (THDR), dihydrodaidzein reductase (DHDR), and daidzein reductase (DZNR)) as well partial downstream and upstream genes based on amino acid sequences. The respective accession numbers were obtained from the National Center for Biotechnology Information (NCBI, https://www.ncbi.nlm.nih.gov/, (accessed on 14 August 2019)) and aligned using clustalW algorithm in the CLC Sequence Viewer software (version 8, Qiagen).

**Alignment and comparison of the equol gene cluster.** The annotated genes of the equol gene cluster of *S*. *isoflavoniconvertens* DSM 22006^T^ and the respective homologue sequences of the above-listed strains were aligned and compared (Figure 3). Detailed information about each annotated nucleotide sequence is given in Supplemental Tables S1–S12. The genes of the equol gene cluster, the position and orientation of genes as well as the upstream and downstream genes are conserved. Noticeably, *S. equolifaciens* DSM 24851^T^ and *Eggerthella* sp. YY7918 showed both the same insertion mutation of a NAD kinase (DMP06_RS06160 and EGYY_15680) and an interchange mutation of the genes *hydG* and EGYY_15670 with *hydE* and EGYY_15660, respectively.

The complete equol gene cluster was identified both in the already known equol-producing strains as well as in *S. faecalis* KGMB04844^T^ and ‘*H*. *massiliensis*’ DSM 101782^T^ which supported the hypothesis that these strains could be able to metabolize daidzein and genistein.

It is not yet clear if the ability of daidzein and genistein metabolization is family, species, or strain dependent [6]. Interestingly, three type strains of the genus *Adlercreutzia*, i.e., *A. equolifaciens* subsp. *equolifaciens* DSM 19450^T^, *A. equolifaciens* subsp. *celatus* DSM 18785^T^, and *A. mucosicola* DSM 19490^T^ harbored the equol gene cluster, whereas the other three type strains of this genus, i.e., *A. caecimuris* DSM 21839^T^, *A. muris* DSM 29508^T^ and *A. rubneri* ResAG-91^T^ did not. Our results are in good agreement with the study of [35], which described the presence of the complete equol operon in the genomes of *A. equolifaciens* subsp. *equolifaciens* DSM 19450^T^ and *A. equolifaciens* subsp. *celatus* DSM 18785^T^, and the absence of this operon in the genome of strain ResAG-91^T^. The incapacity of strain *A. caecimuris* DSM 21839^T^ to metabolize daidzein was already described by [38]. In addition, *A. rubneri* ResAG-91^T^ did neither metabolize daidzein nor genistein which was investigated within this study (see below). [35] described that strain IPLA37004, which belongs to the well-known equol-producing species *A. equolifaciens*, did not produce equol and suggested that this was caused by a deletion in the equol operon. However, results of digital DNA-DNA hybridization using TYGS [39] showed that the similarity of the genome sequence of strain IPLA37004 (GCA_009874275.1) to related type strains of the genus *Adlercreutzia* is below the 70% threshold level for species delineation (results not shown) and therefore, the non-equol-producing strain IPLA37004 represents a potentially new species of *Adlercreutzia.*

**Figure 4:**
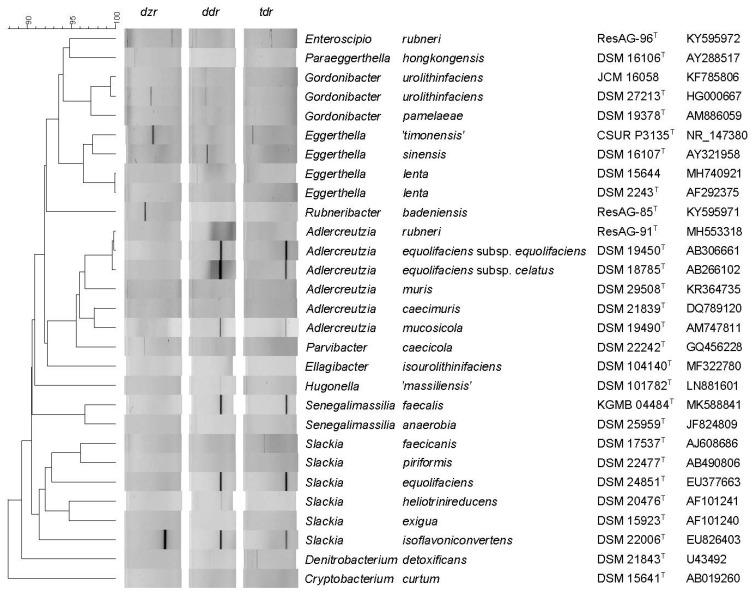
Phylogenetic tree based on 16S rRNA gene sequences of a total of 29 strains belonging to the *Eggerthellaceae* and *Coriobacteriaceae*. Clustering was performed based on multiple alignment and unweighted pair group method with arithmetic mean (UPGMA). Results of a PCR with primers amplifying for *dzr*, *ddr* and *tdr* [29] are shown next to the cluster.

**PCR method to detect *dzr*, *ddr*, and *tdr* genes.** To test the presence of nucleotide sequences coding for *dzr, ddr,* and *tdr* via PCR, primers designed by [29] were used for a total of 29 *Eggerthellaceae* and *Coriobacteriaceae* strains. The results are presented in Figure 4. The amplicon sizes of 203 bp (*dzr*), 205 bp (*ddr*), and 112 bp (*tdr*) as proposed by [29] were confirmed in our study. As already described by [29], the primer for *dzr* amplification did not amplify with the DNA of *S. equolifaciens* DSM 24851^T^ as a template. Therefore, an amplicon of *dzr* could only be detected in the positive control *S*. *isoflavoniconvertens* DSM 22006^T^. The primers for amplification of *ddr* (the gene coding for DHDR) led to products of the expected amplicon size in all previously described equol-producing strains: *A*. *equolifaciens* subsp. *equolifaciens* DSM 19450^T^, *A*. *equolifaciens* subsp. *celatus* DSM 18785^T^, *A*. *mucosicola* DSM 19490^T^, *S*. *isoflavoniconvertens* DSM 22006^T^, and *S*. *equolifaciens* DSM 24851^T^. In addition, a *ddr* amplicon of the expected size was also detected in the strains ‘*H*. *massiliensis*’ DSM 101782^T^ and *S*. *faecalis* KGMB04844^T^. Concerning the presence of the genes coding for THDR*,* putative *tdr* amplicons were detected in *A*. *equolifaciens* subsp. *equolifaciens* DSM 19450^T^, *A*. *equolifaciens* subsp. *celatus* DSM 18785^T^, *A*. *mucosicola* DSM 19490^T^, *S*. *isoflavoniconvertens* DSM 22006^T^, *S*. *equolifaciens* DSM 24851^T^, and *S*. *faecalis* KGMB 04844^T^.

**Investigations of the metabolism of daidzein and genistein.** The same 29 *Eggerthellaceae* and *Coriobacteriaceae* strains were screened for their ability to metabolize daidzein and genistein. For quantification of daidzein, genistein, and their derived metabolites, validated extraction- and LC-DAD-methods were used.

The concentrations at the time points 0, 24, 48, and 72 h of daidzein, genistein, and their derived metabolites in fermentation samples inoculated with strains that metabolized at least either daidzein or genistein are shown in Table 1 and Table 2, respectively. In the control samples without bacteria but supplemented with isoflavones, the initial concentration of 78.4 µM remained stable during the entire course of the fermentation both for daidzein and genistein, and no metabolites were observed. As expected, the metabolization of daidzein to DH-daidzein was conducted by *A*. *equolifaciens* subsp. *celatus* DSM 18785^T^, *A*. *equolifaciens* subsp. *equolifaciens* DSM 19450^T^, *A*. *mucosicola* DSM 19490^T^, *S*. *equolifaciens* DSM 24851^T^, and *S*. *isoflavoniconvertens* DSM 22006^T^. In addition, ‘*H*. *massiliensis*’ DSM 101782^T^ and *S. faecalis* KGMB 04484^T^ proved DH-daidzein production. DH-daidzein was detected after a period of 24 h in all fermentation samples inoculated with the above-listed strains. Furthermore, all strains that metabolized daidzein to DH-daidzein also metabolized DH-daidzein to equol within 24 h except *S. faecalis* KGMB 04484^T^, for which no equol production was observed at no time points. It was unexpected that no equol-production for *S. faecalis* KGMB 04484^T^ was observed since this strain possesses the equol gene cluster. The presence of DH-daidzein in the fermentations inoculated with *S. faecalis* KGMB 04484^T^ was also very low. Therefore, it cannot be ruled out that *S. faecalis* KGMB 04484^T^ is capable to produce equol under different incubation conditions.

As already described in the literature [8,13,14,15,17], the daidzein metabolizing capacity of strains *A*. *equolifaciens* subsp. *celatus* DSM 18785^T^, *A*. *equolifaciens* subsp. *equolifaciens* DSM 19450^T^, *A*. *mucosicola* DSM 19490^T^, *S*. *equolifaciens* DSM 24851^T^, and *S*. *isoflavoniconvertens* DSM 22006^T^ was confirmed within this study. Moreover, the incapacity of strains *S. faecicanis* DSM 17537^T^, *E. lenta* DSM 2243^T^, *P. hongkongensis* DSM 16106^T^, *E. sinensis* DSM 16107^T^, *S. exigua* DSM 15923^T^, and *S. heliotrinireducens* DSM 20476^T^ to metabolize daidzein to equol as already reported by [14] was confirmed by this study. The same strains, i.e., *A*. *equolifaciens* subsp. *celatus* DSM 18785^T^, *A*. *equolifaciens* subsp. *equolifaciens* DSM 19450^T^, *A*. *mucosicola* DSM 19490^T^, *S. faecalis* KGMB 04484^T^, *S*. *equolifaciens* DSM 24851^T^, and *S*. *isoflavoniconvertens* DSM 22006^T^ as well as ‘*H*. *massiliensis*’ DSM 101782^T^ that showed the ability to metabolize daidzein to DH-daidzein also metabolized genistein to DH-genistein. Noteworthy, we observed a U-shaped course of concentrations of daidzein as well as of genistein over time in fermentation samples inoculated with *A. equolifaciens* subsp. *celatus* DSM 18785^T^ (Table 1 and Table 2). There were no hints for artifacts, however, these results cannot be explained and need further investigations.

Under the conditions used in this study, *S*. *exigua* DSM 15923^T^ was the only strain that metabolized daidzein to *O*-DMA which was already reported by [8,14]. However, it has to be noted that in samples of ‘*H*. *massiliensis*’ DSM 101782^T^ small peaks around the retention time of *O*-DMA were detected from 24 h onwards. However, the formation of *O*-DMA could not be shown with certainty. Even if the detected peaks represent *O*-DMA, the produced amounts were around the LOD and thus less than 0.1% of the daidzein added at the beginning of the incubations (data not shown). Further analyses using more sensitive methods should be conducted to elucidate the presence of *O*-DMA in fermentation samples of ‘*H*. *massiliensis*’ DSM 101782^T^. In addition, our study showed that *S*. *exigua* DSM 15923^T^ is also capable to metabolize genistein to 6′-OH-*O*-DMA which was not found by [8]. It has to be noted that under the conditions used in this study, no other investigated strain produced 6′-OH-*O*-DMA in detectable amounts. Interestingly, neither DH-daidzein nor DH-genistein was detected in fermentation samples inoculated with *S*. *exigua* DSM 15923^T^. This leads to the assumption that *O*-DMA and 6′-OH-*O*-DMA is formed without the production of the intermediate compounds DH-daidzein and DH-genistein, respectively. This result is in line as no gene sequences homolog to DZNR could be found in the publicly available genomes of *S. exigua*. A recent study using lactic acid bacterial and bifidobacterial strains showed also that genistein was metabolized to 6′-OH-*O*-DMA without detecting DH-genistein, although daidzein was transformed to *O*-DMA and TH-daidzein alongside the production of DH-daidzein [40].

Interestingly, three strains metabolized genistein to an unknown metabolite which was present after 24 h of sampling and thereafter: *A*. *equolifaciens* subsp. *celatus* DSM 18785^T^, *A. mucosicola* DSM 19490^T^ and ‘*H*. *massiliensis*’ DSM 101782^T^ converted genistein into DH-genistein and an unknown metabolite at a retention time of 3.3 min. In addition, fermentation samples inoculated with *A*. *equolifaciens* subsp. *equolifaciens* DSM 19450^T^ showed the same unknown metabolite from 48 h on. The UV spectra of this unknown metabolite exhibited a maximum absorption at 275 nm and showed similarities with the UV spectrum of the daidzein metabolite equol with a maximum absorption at 280 nm (data not shown). Thus, this metabolite is very likely to be 5-OH-equol. Calibration curves of the reference compound equol were used to estimate the amount of this metabolite. The production of 5-OH-equol was highest in strain ‘*H*. *massiliensis*’ DSM 101782^T^, followed by *A. mucosicola* DSM 19490^T^. The formation of 5-OH-equol in strains *A*. *equolifaciens* subsp. *equolifaciens* DSM 19450^T^ and *A*. *equolifaciens* subsp. *celatus* DSM 18785^T^ were comparable but notably lower.

**LC-MS analysis of the unknown microbial genistein metabolite.** To characterize this unknown metabolite, fermentation samples of genistein inoculated with ‘*H*. *massiliensis*’ DSM 101782^T^ at time points 0 h and 72 h were analysed by high-resolution LC-MS. The unknown metabolite peak in the 72 h fermentation sample eluted a bit earlier in the LC-MS analysis (other HPLC device with different dead volume) and exhibited in full-scan MS a mass/charge ratio (*m*/*z*) of 259.0972 and *m*/*z* 257.0829 in the positive and negative ionization mode, respectively. These *m*/*z* values concurred with the chemical formula of 5-OH-equol (C_15_H_14_O_4_, mass error 2.8 ppm and 3.8 ppm in positive and negative mode, respectively). The positive MS/MS spectrum of the precursor ion 259.1 of the unknown metabolite peak exhibited the following characteristic fragment ions (*m*/*z*) 165.0547, 153.0545, 139.0381, 133.0647, 121.028, and 107.048. This matched the MS/MS spectrum of 5-OH-equol described by [9]. LC-MS measurements of *A*. *mucosicola* DSM 19490^T^ 72 h-sample supplemented with genistein revealed similar results.

Conclusively, the unknown metabolite peak in fermentation samples of genistein inoculated with *A*. *mucosicola* DSM 19490^T^ and ‘*H*. *massiliensis*’ DSM 101782^T^ was putatively identified as 5-OH-equol. Due to the lack of a reference standard for 5-OH-equol, the final confirmation of the identity as well as the quantitation in the samples should be conducted in further studies. The genistein fermentation samples of *A*. *equolifaciens* subsp. *celatus* DSM 18785^T^ and *A*. *equolifaciens* subsp. *equolifaciens* DSM 19450^T^ were not measured by LC-MS within this study. Nevertheless, the results of the LC-DAD analysis (retention time and UV spectra) led to the assumption that the unknown peak in fermentation samples of *A*. *equolifaciens* subsp. *celatus* DSM 18785^T^ and of *A*. *equolifaciens* subsp. *equolifaciens* DSM 19450^T^ represents also 5-OH-equol. It must be noted that the type strain of *S*. *isoflavoniconvertens* DSM 22006^T^ was described to be capable of 5-OH-equol production in the original strain description [8], although this metabolite could not be detected in genistein fermentation samples inoculated with this strain under comparable conditions in this study, e.g., medium, gas atmosphere, temperature, concentration of isoflavone supplementation.

## 4. Conclusions

Amino acid sequences comprising genes coding for daidzein reductase (DZNR), dihydrodaidzein reductase (DHDR), tetrahydrodaidzein reductase (THDR) and dihydrodaidzein racemase (DDRC) were successfully used to search for similar protein sequences within draft genome sequences of *Eggerthellaceae* and *Coriobacteriaceae* strains. Homolog genes of the equol gene cluster were detected and aligned in already described equol-producing strains. Furthermore, this cluster was newly detected in strains of the *Eggerthellaceae* and *Coriobacteriaceae* that have so far not been associated with equol production. The presence of genes of the equol gene cluster was confirmed via PCR amplification. In addition, the metabolism of daidzein and genistein was investigated using pure cultures of *Eggerthellaceae* and *Coriobacteriaceae* strains. In conclusion, this study led to the first description of the human gut bacterial strains ‘*Hugonella massiliensis’* DSM 101782^T^ and *Senegalimassilia faecalis* KGMB 04484^T^ as capable of metabolizing daidzein and genistein.

## Figures and Tables

**Table 1:** Concentrations [µM] of daidzein and its metabolites measured by LC-DAD in fermentation samples inoculated with pure cultures of *Eggerthellaceae* and *Coriobacteriaceae* strains. The initial concentration of daidzein was 78.4 µM. Results are the mean values ± standard deviations of triplicates and for their calculation, values between limit of detection (LOD) and limit of quantitation (LOQ) were set as LOD, and values <LOD were set as zero. DH-Daidzein, Dihydrodaidzein; *O*-DMA, *O*-Desmethylangolensin; SD, standard deviation; -, not detected or values below LOD.

	T (h)	Daidzein	DH-Daidzein	Equol	*O*-DMA
Control (medium without bacteria) ^#^	0	75.47 ± 5.05	-	-	-
	24	77.54 ± 3.05	-	-	-
	48	79.11 ± 0.59	-	-	-
	72	78.13 ± 2.04	-	-	-
*Adlercreutzia equolifaciens* subsp. *celatus* DSM 18785^T^	0	77.80 ± 1.31	-	-	-
	24	5.04 ± 4.44	18.49 ± 11.57	44.00 ± 4.41	-
	48	15.70 ± 3.00	23.15 ± 1.78	29.08 ± 4.13	-
	72	61.61 ± 4.49	10.99 ± 1.70	2.58 ± 1.31	-
*Adlercreutzia equolifaciens* subsp. *equolifaciens* DSM 19450^T^	0	72.48 ± 7.86	-	-	-
	24	24.03 ± 3.55	26.23 ± 1.48	19.39 ± 1.08	-
	48	2.06 ± 0.73	11.02 ± 2.56	51.09 ± 3.75	-
	72	6.04 ± 3.87	26.80 ± 1.10	32.77 ± 4.37	-
*Adlercreutzia mucosicola* DSM 19490^T^	0	78.68 ± 2.69	-	-	-
	24	in three samples *	0.41 ± 0.62	64.55 ± 0.79	-
	48	in three samples *	0.48 ± 0.74	64.55 ± 0.58	-
	72	1.79 ± 0.32	2.23 ± 0.59	62.70 ± 0.28	-
‘*Hugonella massiliensis*’ DSM 101782^T^	0	77.20 ± 1.32	-	-	-
	24	-	in three samples *	63.44 ± 4.60	-
	48	in three samples *	in three samples *	64.93 ± 1.89	-
	72	in three samples *	in three samples *	64.61 ± 2.14	-
*Senegalimassilia faecalis* KGMB 04484^T^	0	52.36 ± 28.73	-	-	-
	24	69.24 ± 4.44	in one sample *	-	-
	48	68.36 ± 1.98	in one sample *	-	-
	72	66.52 ± 2.35	in two samples *	-	-
*Slackia equolifaciens* DSM 24851^T #^	0	67.70 ± 8.34	-	-	-
	24	52.34 ± 3.73	18.11 ± 1.40	5.42 ± 1.72	-
	48	19.89 ± 3.87	33.91 ± 0.51	16.49 ± 3.46	-
	72	22.23 ± 4.51	32.70 ± 0.21	15.48 ± 3.62	-
*Slackia exigua* DSM 15923^T^	0	79.09 ± 0.70	-	-	-
	24	22.51 ± 2.11	-	-	42.20 ± 1.91
	48	24.16 ± 3.38	-	-	43.29 ± 2.19
	72	23.25 ± 3.64	-	-	43.44 ± 2.24
*Slackia isoflavoniconvertens* DSM 22006^T^	0	78.27 ± 3.82	-	-	-
	24	11.70 ± 8.46	17.35 ± 5.72	37.79 ± 4.21	-
	48	1.29 ± 0.19	10.95 ± 3.65	52.32 ± 5.25	-
	72	4.73 ± 1.16	17.88 ± 1.37	43.72 ± 3.49	-

* Detected values ≥ LOD, but below LOQ. **^#^** Reported values are from one representative experiment (*n* = 3).

**Table 2:** Concentrations [µM] of genistein and its metabolites measured by LC-DAD in fermentation samples inoculated with pure cultures of *Eggerthellaceae* and *Coriobacteriaceae* strains. The initial concentration of genistein was 78.4 µM. Results are the mean values ± standard deviations of triplicates, except results labeled with ^§^ which are in duplicate. For their calculation, values between limit of detection (LOD) and limit of quantitation (LOQ) were set as LOD, and values <LOD were set as zero. DH-Genistein, Dihydrogenistein; 6′OH-*O*-DMA, 6′-Hydroxy-*O*-desmethylangolensin; 5-OH-Equol, 5-Hydroxy-equol; SD, standard deviation; -, not detected or values below LOD.

	T (h)	Genistein	DH-Genistein	5-OH-Equol	6′OH-*O*-DMA
Control (medium without bacteria) ^#^	0	81.17 ± 0.80	-	-	-
	24	79.05 ± 1.44	-	-	-
	48	74.91 ± 4.86	-	-	-
	72	75.78 ± 3.52	-	-	-
*Adlercreutzia equolifaciens* subsp. *celatus* DSM 18785^T^	0	78.21 ± 9.11	-	-	-
	24	4.32 ± 5.06	59.40 ± 0.81	2.05 ± 1.65	-
	48	14.67 ± 1.85	48.38 ± 3.19	1.78 ± 1.43	-
	72	42.33 ± 6.17	25.88 ± 6.96	1.49 ± 1.15	-
*Adlercreutzia equolifaciens* subsp. *equolifaciens* DSM 19450^T^	0	78.17 ± 6.66	-	-	-
	24	29.04 ± 26.03	41.61 ± 19.58	-	-
	48	2.15 ± 0.92	59.16 ± 1.17	1.42 ± 1.10	-
	72	14.99 ± 5.16	45.90 ± 4.00	1.83 ± 0.22	-
*Adlercreutzia mucosicola* DSM 19490^T^	0	79.21 ± 1.15 ^§^	- ^§^	- ^§^	- ^§^
	24	0.75 ± 0.99 ^§^	48.76 ± 7.63 ^§^	10.02 ± 3.54 ^§^	- ^§^
	48	0.72 ± 0.95 ^§^	26.77 ± 10.20 ^§^	20.17 ± 4.81 ^§^	- ^§^
	72	7.22 ± 0.58 ^§^	16.89 ± 9.19 ^§^	20.33 ± 4.20 ^§^	- ^§^
‘*Hugonella massiliensis*’ DSM 101782^T^	0	84.05 ± 2.44	-	-	-
	24	1.94 ± 3.27	16.99 ± 14.19	27.36 ± 10.17	-
	48	in one sample *	0.91 ± 1.48	33.93 ± 2.40	-
	72	in one sample *	in three samples *	32.28 ± 2.80	-
*Senegalimassilia faecalis* KGMB 04484^T^	0	90.96 ± 4.59	-	-	-
	24	67.93 ± 8.13	-	-	-
	48	71.16 ± 0.37	-	-	-
	72	71.57 ± 1.36	in three samples *	-	-
*Slackia equolifaciens* DSM 24851^T #^	0	70.47 ± 7.56	-	-	-
	24	42.99 ± 2.42	29.83 ± 1.63	-	-
	48	25.58 ± 0.99	42.12 ± 1.65	-	-
	72	34.66 ± 0.22	33.97 ± 0.33	-	-
*Slackia exigua* DSM 15923^T^	0	73.80 ± 3.44	-	-	-
	24	31.44 ± 10.95	-	-	12.74 ± 1.23
	48	10.14 ± 12.55 ^§^	- ^§^	- ^§^	5.08 ± 0.68 ^§^
	72	20.52 ± 16.91 ^§^	- ^§^	- ^§^	- ^§^
*Slackia isoflavoniconvertens* DSM 22006^T^	0	75.31 ± 6.70 ^§^	- ^§^	- ^§^	- ^§^
	24	36.60 ± 12.03	39.13 ± 9.73	-	-
	48	7.17 ± 2.25	59.27 ± 1.68	-	-
	72	9.13 ± 0.41	55.71 ± 0.11	-	-

* Detected values ≥ LOD, but below LOQ. **^#^** Reported values are from one representative experiment (*n* = 3).

## Supplementary Materials

The following are available online at https://www.mdpi.com/article/10.3390/foods10112741/s1, Tables S1: Annotation of the conserved open reading frames (orfs) within the equol biosynthesis gene cluster of *Slackia isoflavoniconvertens* DSM 22006^T^ (GenBank: JQ358709), Tables S2: Annotation of the conserved open reading frames (orfs) within the equol biosynthesis gene cluster of *Slackia isoflavoniconvertens* DSM 22006^T^ (NCBI reference sequence: NZ_QIBZ01000017.1), Tables S3: Annotation of the conserved open reading frames (orfs) within the equol biosynthesis gene cluster of *Slackia equolifaciens* DSM 24851^T^, Tables S4: Annotation of the conserved open reading frames (orfs) within the equol biosynthesis gene cluster of *Eggerthella sp*. YY918, Tables S5: Annotation of the conserved open reading frames (orfs) within the equol biosynthesis gene cluster of *Adlercreutzia equolifaciens* subsp. equolifaciens DSM 19450^T^, Tables S6: Annotation of the conserved open reading frames (orfs) within the equol biosynthesis gene cluster of *Adlercreutzia equolifaciens* subsp. celatus DSM 18785^T^, Tables S7: Annotation of the conserved open reading frames (orfs) within the equol biosynthesis gene cluster of *Adlercreutzia mucosicola* DSM 19490^T^, Tables S8: Annotation of the conserved open reading frames (orfs) within the equol biosynthesis gene cluster of *Senegalimassilia faecalis* KGMB04484^T^, Tables S9: Annotation of the conserved open reading frames (orfs) within the equol biosynthesis gene cluster of ‘*Hugonella massiliensis’* AT8^T^, Tables S10: Annotation of the conserved open reading frames (orfs) within the equol biosynthesis gene cluster of *Lactococcus garvieae* 20-92, Tables S11: Annotation of the conserved open reading frames (orfs) within the equol biosynthesis gene cluster of *Slackia sp*. AUH-JLC159, Tables S12: Annotation of the conserved open reading frames (orfs) within the equol biosynthesis gene cluster of *Slackia sp.* NATTS, Data S13: Results of validation (accuracy, intra-day precision, recovery, LOD, LOQ and linearity) of LC-DAD analyses of daidzein, genistein and corresponding microbial metabolites in fermentation samples of pure cultures.
