# Supplementary material for: Metabolism of Daidzein and Genistein by Gut Bacteria of the Class Coriobacteriia
Source: Foods. 2021 Nov 9;10(11):2741. doi: 10.3390/foods10112741 (PMC8618169; doi:10.3390/foods10112741)
Supplement: Supplementary file 1 [file foods-10-02741-s001.zip › foods-1424178-supplementary.pdf]

Supplemental Table S1: Annotation of the conserved open reading frames (orfs) within the equol biosynthesis gene cluster of ***Slackia isoflavoniconvertens* DSM 22006**[T] (GenBank: JQ358709).

Locus_tag	Strand	Position[a]	Gene product	Protein ID [sequence length]
**ifcA**	+	374-847	hypothetical protein	AFV15447.1 [157 aa]
**ifcB**	+	866-1588	putative electron transfer flavoprotein beta-subunit	AFV15448.1 [240 aa]
**ifcC**	+	1588-2496	putative electron transfer flavoprotein alpha-subunit	AFV15449.1 [302 aa]
**tdr**	+	2572-4032	tetrahydrodaidzein reductase	AFV15450.1 [486 aa]
**ddr**	+	4169-5026	dihydrodaidzein reductase	AFV15451.1 [285 aa]
**ifcD**	+	5150-5635	hypothetical protein	AFV15452.1 [161 aa]
**dzr**	+	5690-7624	daidzein reductase	AFV15453.1 [644 aa]

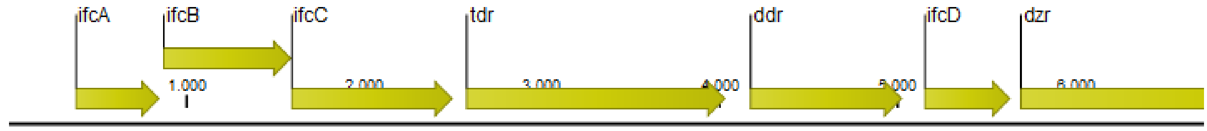

[a], *Slackia isoflavoniconvertens* hypothetical protein (*ifcA*), putative electron transfer flavoprotein beta-subunit (*ifcB*), putative electron transfer flavoprotein alpha-subunit (*ifcC*), tetrahydrodaidzein reductase (*tdr*), dihydrodaidzein reductase (*ddr*), hypothetical protein (*ifcD*), and daidzein reductase (*dzr*) genes, complete cds. GenBank: JQ358709.

Supplemental Table S2: Annotation of the conserved open reading frames (orfs) within the equol biosynthesis gene cluster of ***Slackia isoflavoniconvertens* DSM 22006**[T] (NCBI reference sequence: NZ_QIBZ01000017.1).

Locus_tag	Strand	Position[a]	Gene product	Protein ID [sequence length]
**DMP05_RS08425**	-	19422-20795	ATP-binding protein	WP_123220029.1 [457 aa]
**DMP05_RS08430**	-	21341-22750	[FeFe] hydrogenase H-cluster maturation GTPase HydF	WP_123220030.1 [469 aa]
**DMP05_RS08435**	-	22824-24275	[FeFe] hydrogenase H-cluster radical SAM maturase HydG	WP_123220031.1 [483 aa]
**DMP05_RS08440**	-	24300-25364	[FeFe] hydrogenase H-cluster radical SAM maturase HydE	WP_123220054.1 [354 aa]
**DMP05_RS08445**	-	25384-27102	hydrogenase	WP_123220032.1 [572 aa]
**DMP05_RS08450**	-	27095-28948	NAD(P)-binding protein	WP_123220033.1 [617 aa]
**DMP05_RS08455**	+	29304-29423	SDR family oxidoreductase	WP_123220055.1 [39 aa]
**DMP05_RS08460**	-	29595-31529	FAD-dependent oxidoreductase	WP_123220034.1 [644 aa]
**DMP05_RS08465**	-	31584-32069	hypothetical protein	WP_123220035.1 [161 aa]
**DMP05_RS08470**	-	32193-33050	SDR family oxidoreductase	WP_123220036.1 [285 aa]
**DMP05_RS08475**	-	33187-34647	FAD-dependent oxidoreductase	WP_123220037.1 [486 aa]
**DMP05_RS08480**	-	34723-35631	electron transfer flavoprotein subunit alpha/FixB family protein	WP_123220038.1 [302 aa]
**DMP05_RS08485**	-	35631-36353	electron transfer flavoprotein subunit alpha	WP_123220039.1 [240 aa]
**DMP05_RS08490**	-	36372-36845	glyoxalase	WP_123220056.1 [157 aa]

**DMP05_RS08495**	-	37202-37891	response regulator transcription factor	WP_123220057.1 [229 aa]

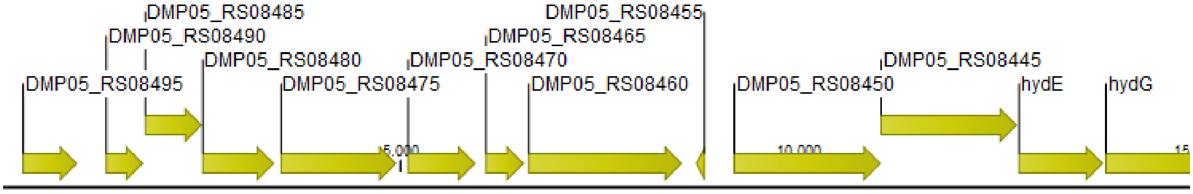

[a], *Slackia isoflavoniconvertens* strain DSM 22006 DSM22006T_contig_017, whole genome shotgun sequence. NCBI Reference Sequence: NZ_QIBZ01000017.1. Representation in reverse-complement.

Supplemental Table S3: Annotation of the conserved open reading frames (orfs) within the equol biosynthesis gene cluster of ***Slackia equolifaciens* DSM 24851^T.**

**Locus_tag**	**Strand**	**Position[a]**	**Gene product**	**Protein ID [sequence length]**
**DMP06_RS06145**	-	33322-34662	[FeFe] hydrogenase H-cluster maturation GTPase HydF	WP_123208874.1 [446 aa]
**DMP06_RS06150**	-	34719-35780	[FeFe] hydrogenase H-cluster radical SAM maturase HydE	WP_123208875.1 [353 aa]
**DMP06_RS06155**	-	35791-37215	[FeFe] hydrogenase H-cluster radical SAM maturase HydG	WP_123208876.1 [474 aa]
**DMP06_RS06160**	-	37246-38157	NAD(+)/NADH kinase	WP_123208877.1 [303 aa]
**DMP06_RS06165**	-	38301-40019	hydrogenase	WP_123208878.1 [572 aa]
**DMP06_RS06170**	-	40013-41851	NAD(P)-binding protein	WP_123208879.1 [612 aa]
**DMP06_RS06175**	-	41872-42177	ferredoxin family protein	WP_123208880.1 [101 aa]
**DMP06_RS06180**	-	42174-43487	FAD-dependent oxidoreductase	WP_123208881.1 [437 aa]
**DMP06_RS06185**	-	43689-45623	FAD-binding protein	WP_123208882.1 [644 aa]
**DMP06_RS06190**	-	45696-46199	hypothetical protein	WP_123208883.1 [167 aa]
**DMP06_RS06195**	-	46296-47228	SDR family oxidoreductase	WP_123208884.1 [310 aa]
**DMP06_RS06200**	-	47302-48762	FAD-dependent oxidoreductase	WP_123208885.1 [486 aa]
**DMP06_RS06205**	-	48874-49788	electron transfer flavoprotein subunit alpha/FixB family protein	WP_013979961.1 [304 aa]
**DMP06_RS06210**	-	49789-50511	electron transfer flavoprotein beta-subunit	WP_013979962.1 [240 aa]
**DMP06_RS06215**	-	50527-51003	hypothetical protein	WP_013979963.1 [158 aa]
**DMP06_RS06220**	-	51337-52116	response regulator transcription factor	WP_123208886.1 [259 aa]

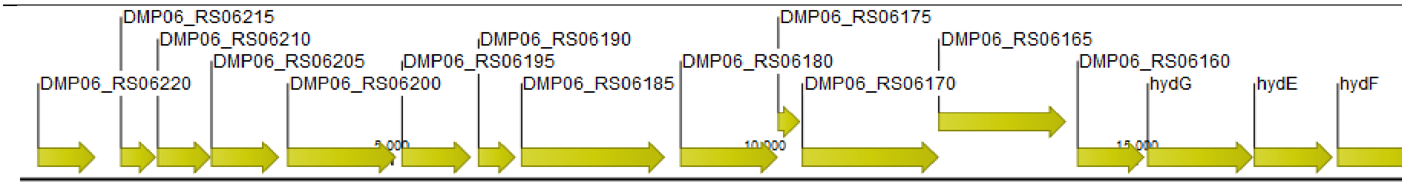

[a], *Slackia equolifaciens* strain DSM 24851 DSM24851T_contig_009, whole genome shotgun sequence. NCBI Reference Sequence: NZ_QIBX01000009.1. Representation in reverse-complement.

Supplemental Table S4: Annotation of the conserved open reading frames (orfs) within the equol biosynthesis gene cluster of ***Eggerthella* sp. YY918.**

Locus_tag	Strand	Position[a]	Gene product	Protein ID [sequence length]
**EGYY_15650**	-	1675997-1677337	hypothetical protein	BAK44705.1 [446 aa]
**EGYY_15660**	-	1677394-1678455	hypothetical protein	BAK44706.1 [353 aa]
**EGYY_15670**	-	1678466-1679890	hypothetical protein	BAK44707.1 [474 aa]
**EGYY_15680**	-	1679921-1680802	hypothetical protein	BAK44708.1 [393 aa]
**EGYY_15690**	-	1680976-1682694	hypothetical protein	BAK44709.1 [572 aa]
**EGYY_15700**	-	1682688-1684526	hypothetical protein	BAK44710.1 [612 aa]
**EGYY_15710**	-	1684547-1684852	ferredoxin-like protein	BAK44711.1 [101 aa]
**EGYY_15720**	-	1684849-1686162	dehydrogenase	BAK44712.1 [437 aa]
**EGYY_15730**	-	1686364-1688298	NADH:flavin oxidoreductase	BAK44713.1 [644 aa]
**EGYY_15740**	-	1688371-1688871	predicted outer membrane protein	BAK44714.1 [166 aa]
**EGYY_15750**	-	1688990-1689850	hypothetical protein	BAK44715.1 [286 aa]
**EGYY_15760**	-	1689996-1691456	hypothetical protein	BAK44716.1 [486 aa]
**EGYY_15770**	-	1691568-1692482	electron transfer flavoprotein alpha-subunit	BAK44717.1 [304 aa]
**EGYY_15780**	-	1692483-1693205	electron transfer flavoprotein beta-subunit	BAK44718.1 [240 aa]
**EGYY_15790**	-	1693221-1693697	hypothetical protein	BAK44719.1 [158 aa]
**EGYY_15800**	-	1694030-1694809	hypothetical protein	BAK44720.1 [259 aa]

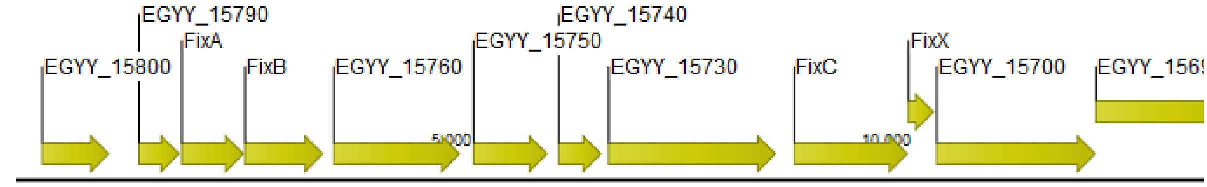

[a], *Eggerthella* sp. YY7918 DNA, complete genome. GenBank: AP012211.1. Representation in reverse-complement.

Supplemental Table S5: Annotation of the conserved open reading frames (orfs) within the equol biosynthesis gene cluster of ***Adlercreutzia equolifaciens* subsp. *equolifaciens* DSM 19450^T.**

**Annotation and open orfs of the equol biosynthesis gene cluster of *Adlercreutzia equolifaciens* subsp. *equolifaciens* DSM 19450^T**				
**Locus_tag**	**Strand**	**Position[a]**	**Gene product**	**Protein ID [sequence length]**
**AEQU_2235**	-	2791670-2792440	two-component response regulator	BAN78204.1 [256 aa]
**AEQU_2234**	-	2791008-2791460	putative_dihydrodaizein_racemase	BAN78203.1 [150 aa]
**AEQU_2233**	-	2790267-2790986	electron transfer flavoprotein beta subunit	BAN78202.1 [239 aa]
**AEQU_2232**	-	2789323-2790237	electron transfer flavoprotein alpha subunit	BAN78201.1 [304 aa]
**AEQU_2231**	-	2787796-2789259	putative_tetrahydrodaidzein_reductase	BAN78200.1 [487 aa]

**AEQU_2230**	-	2786868-2787716	putative_dihydrodaidzein_reductase	BAN78199.1 [282 aa]
**AEQU_2229**	-	2786295-2786774	hypothetical protein	BAN78198.1 [159 aa]
**AEQU_2228**	-	2784304-2786232	putative_daidzein_reductase	BAN78197.1 [642 aa]
**AEQU_2227**	-	2782920-2784233	flavin-dependent dehydrogenase	BAN78196.1 [437 aa]
**AEQU_2226**	-	2782612-2782923	putative ferredoxin	BAN78195.1 [103 aa]
**AEQU_2225**	-	2780557-2782395	putative glutamate synthase	BAN78194.1 [612 aa]
**AEQU_2224**	-	2778770-2780563	dehydrogenase	BAN78193.1 [597 aa]
**AEQU_2223**	-	2777598-2778668	conserved hypothetical protein	BAN78192.1 [356 aa]
**AEQU_2222**	-	2776166-2777611	putative thiamine biosynthesis protein	BAN78191.1 [481 aa]
**AEQU_2221**	-	2774610-2776022	GTP-binding protein	BAN78190.1 [470 aa]

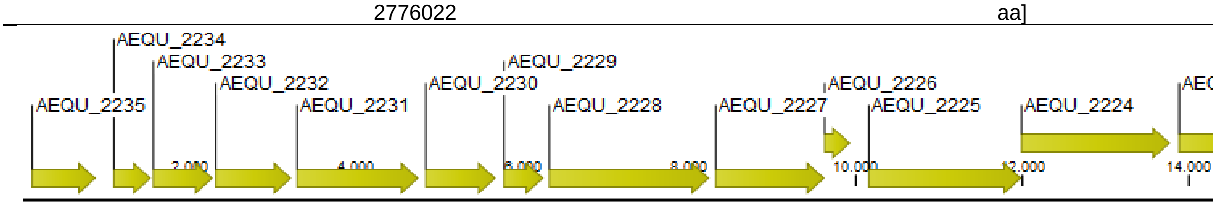

[a], *Adlercreutzia equolifaciens* DSM 19450 DNA, complete genome. GenBank: AP013105.1. Representation in reverse-complement.

Supplemental Table S6: Annotation of the conserved open reading frames (orfs) within the equol biosynthesis gene cluster of ***Adlercreutzia equolifaciens*** **subsp.** ***celatus*** **DSM 18785^{T}.**

**Locus_tag**	**Strand**	**Position[a]**	**Gene product**	**Protein ID [sequence length]**
**DMP10_RS02885**	-	162209-163516	[FeFe] hydrogenase H-cluster maturation GTPase HydF	WP_117283648.1 [435 aa]
**DMP10_RS02890**	-	163661-165103	[FeFe] hydrogenase H-cluster radical SAM maturase HydG	WP_117283649.1 [480 aa]
**DMP10_RS02895**	-	165090-166160	[FeFe] hydrogenase H-cluster radical SAM maturase HydE	WP_117283650.1 [356 aa]
**DMP10_RS02900**	-	166266-168059	2Fe-2S iron-sulfur cluster binding domain-containing protein	WP_117283651.1 [597 aa]
**DMP10_RS02905**	-	168053-169891	NAD(P)-binding protein	WP_117283652.1 [612 aa]
**DMP10_RS02910**	-	170107-170418	ferredoxin family protein	WP_117283653.1 [103 aa]
**DMP10_RS02915**	-	170415-171728	FAD-dependent oxidoreductase	WP_117283654.1 [437 aa]
**DMP10_RS02920**	-	171799-173727	NAD(P)-binding protein	WP_035023034.1 [642 aa]
**DMP10_RS02925**	-	173792-174271	hypothetical protein	WP_022741750.1 [159 aa]
**DMP10_RS02930**	-	174365-175213	SDR family oxidoreductase	WP_117283655.1 [282 aa]
**DMP10_RS02935**	-	175294-176757	FAD-dependent oxidoreductase	WP_117283656.1 [487 aa]
**DMP10_RS02940**	-	176821-177735	electron transfer flavoprotein subunit alpha/FixB family protein	WP_117283657.1 [304 aa]
**DMP10_RS02945**	-	177765-178484	electron transfer flavoprotein subunit alpha	WP_117283658.1 [239 aa]
**DMP10_RS02950**	-	178506-178958	glyoxalase	WP_117283659.1 [150 aa]
**DMP10_RS02955**	-	179168-180001	response regulator transcription factor	WP_117283660.1 [277 aa]

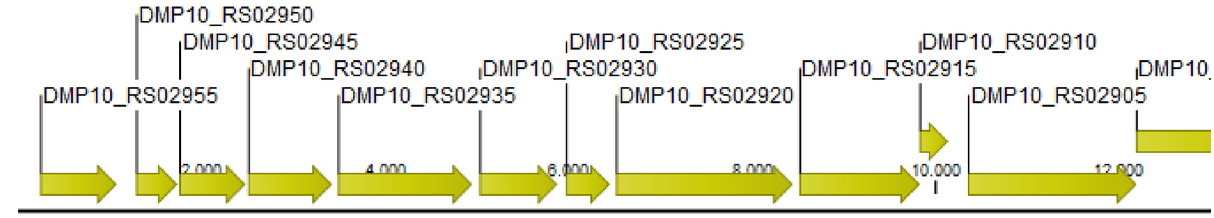

[a], *Asaccharobacter celatus* DSM 18785 DSM18785T_contig_003, whole genome shotgun sequence. GenBank: NZ_QICA01000003.1. Representation in reverse-complement.

Supplemental Table S7: Annotation of the conserved open reading frames (orfs) within the equol biosynthesis gene cluster of ***Adlercreutzia mucosicola* DSM 19490^{T}**.

Locus_tag	Strand	Position[a]	Gene product	Protein ID [sequence length]
**G503_RS0111220**	-	48652-49869	[FeFe] hydrogenase H-cluster maturation GTPase HydF	WP_028027639.1 [405 aa]
**G503_RS0111225**	-	49881-51356	[FeFe] hydrogenase H-cluster radical SAM maturase HydG	WP_051191296.1 [491 aa]
**G503_RS0111230**	-	51316-52392	[FeFe] hydrogenase H-cluster radical SAM maturase HydE	WP_035023031.1 [358 aa]
**G503_RS0111235**	-	52482-54194	hydrogenase	WP_028027642.1 [570 aa]
**G503_RS0111240**	-	54188-56026	FAD-dependent oxidoreductase	WP_028027643.1 [612 aa]
**G503_RS0111245**	-	56238-56549	ferredoxin	WP_028027644.1 [103 aa]
**G503_RS0111250**	-	56546-57859	FAD-dependent oxidoreductase	WP_028027645.1 [437 aa]
**G503_RS0111255**	-	57930-59858	NAD(P)-binding protein	WP_035023034.1 [642 aa]
**G503_RS0111260**	-	59924-60403	hypothetical protein	WP_022741750.1 [159 aa]
**G503_RS14985**	-	60497-61345	SDR family oxidoreductase	WP_051191183.1 [282 aa]
**G503_RS0111270**	-	61426-62931	FAD-dependent oxidoreductase	WP_084636580.1 [501 aa]
**G503_RS0111275**	-	62952-63866	electron transfer flavoprotein subunit alpha/FixB family protein	WP_028027648.1 [304 aa]
**G503_RS0111280**	-	63894-64613	hypothetical protein	WP_028027649.1 [239 aa]
**G503_RS0111285**	-	64632-65084	hypothetical protein	WP_051191185.1 [150 aa]
**G503_RS0111290**	-	65297-66067	response regulator transcription factor	WP_028027651.1 [256 aa]

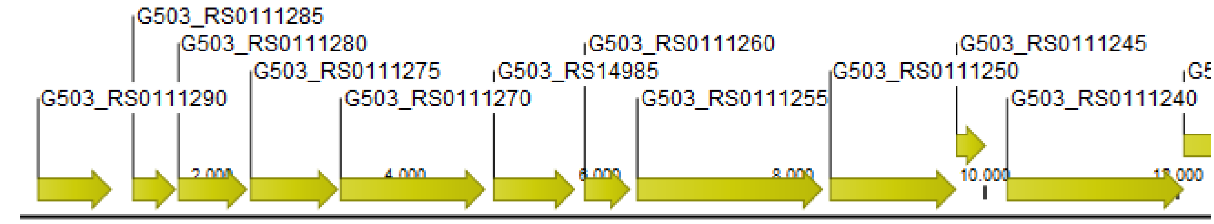

[a], *Enterorhabdus mucosicola* DSM 19490 G503DRAFT_scaffold00001.1, whole genome shotgun sequence, NCBI Reference Sequence: NZ_KE383895.1. Representation in reverse-complement.

Supplemental Table S8: Annotation of the conserved open reading frames (orfs) within the equol biosynthesis gene cluster of ***Senegalimassilia faecalis* KGMB04484^{T}.**

**Locus_tag**	**Strand**	**Position[a]**	**Gene product**	**Protein ID [sequence length]**
**ET524_RS10245**	+	2468498-2469688	ABC transporter permease	WP_129425566.1 [396 aa]
**ET524_RS10250**	+	2469894-2470664	response regulator transcription factor	WP_129425568.1 [256 aa]
**ET524_RS10255**	+	2470964-2471437	glyoxalase	WP_129425570.1 [157 aa]
**ET524_RS10260**	+	2471458-2472180	hypothetical protein	WP_129425572.1 [240 aa]
**ET524_RS10265**	+	2472181-2473095	electron transfer flavoprotein subunit alpha/FixB family protein	WP_129425574.1 [304 aa]
**ET524_RS10270**	+	2473202-2474665	FAD-dependent oxidoreductase	WP_129425576.1 [487 aa]
**ET524_RS10275**	+	2474795-2475658	SDR family oxidoreductase	WP_129425578.1 [287 aa]
**ET524_RS10280**	+	2475780-2476280	hypothetical protein	WP_129425580.1 [166 aa]
**ET524_RS10285**	+	2476353-2478287	NAD(P)-binding protein	WP_129425582.1 [644 aa]
**ET524_RS10290**	+	2478487-2479788	FAD-dependent oxidoreductase	WP_129425584.1 [433 aa]
**ET524_RS10295**	+	2479785-2480084	ferredoxin family protein	WP_129425586.1 [99 aa]
**ET524_RS10300**	+	2480101-2481939	FAD-dependent oxidoreductase	WP_129425588.1 [612 aa]
**ET524_RS10305**	+	2481932-2483650	2Fe-2S iron-sulfur cluster binding domain-containing protein	WP_129425590.1 [572 aa]
**ET524_RS10310**	+	2483651-2484703	[FeFe] hydrogenase H-cluster radical SAM maturase HydE	WP_129425592.1 [350 aa]
**ET524_RS10315**	+	2484700-2486151	[FeFe] hydrogenase H-cluster radical SAM maturase HydG	WP_129425594.1 [483 aa]
**ET524_RS10320**	+	2486163-2487413	[FeFe] hydrogenase H-cluster maturation GTPase HydF	WP_129425596.1 [416 aa]

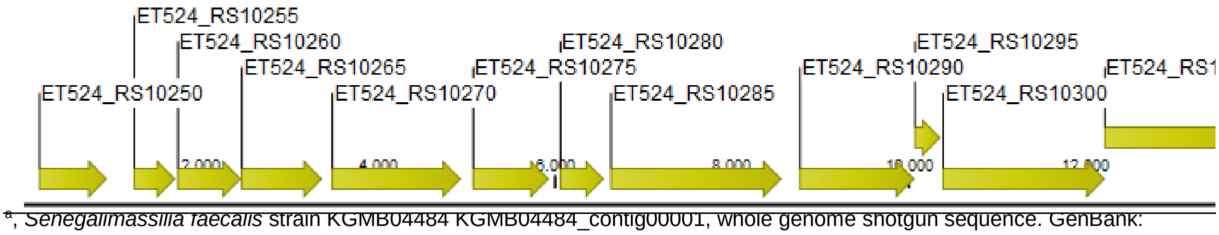

[a], *Senegalimassilia faecalis* strain KGMB04484 KGMB04484_contig00001, whole genome shotgun sequence. GenBank: NZ_SDPW01000001.1

Supplemental Table S9: Annotation of the conserved open reading frames (orfs) within the equol biosynthesis gene cluster of **'*Hugonella massiliensis*' AT8^{T}.**

**Locus_tag**	**Strand**	**Position[a]**	**Gene product**	**Protein ID [sequence length]**
**BN3270_RS00235**	-	10492-11235	response regulator transcription factor	WP_058985114.1 [247 aa]
**BN3270_RS00230**	-	9734-10156	glyoxalase	WP_058985167.1 [140 aa]
**BN3270_RS00225**	-	8987-9730	hypothetical protein	WP_082673216.1 [247 aa]
**BN3270_RS00220**	-	8072-8986	electron transfer flavoprotein subunit alpha/FixB family protein	WP_058985112.1 [304 aa]
**BN3270_RS00215**	-	6536-7999	FAD-dependent oxidoreductase	WP_058985111.1 [487 aa]
**BN3270_RS00210**	-	5607-6467	SDR family oxidoreductase	WP_082673215.1 [286 aa]
**BN3270_RS00205**	-	4978-5460	hypothetical protein	WP_058985110.1 [160 aa]

**BN3270_RS00200**	-	2981-4978	NAD(P)-binding protein	WP_082673214.1 [665 aa]
**BN3270_RS00195**	-	1548-2918	FAD-dependent oxidoreductase	WP_082673213.1 [456 aa]
**BN3270_RS00190**	-	1237-1548	ferredoxin	WP_058985165.1 [103 aa]
**BN3270_RS00185**	-	90-1193	NAD(P)-binding protein	WP_058985107.1 [367 aa]

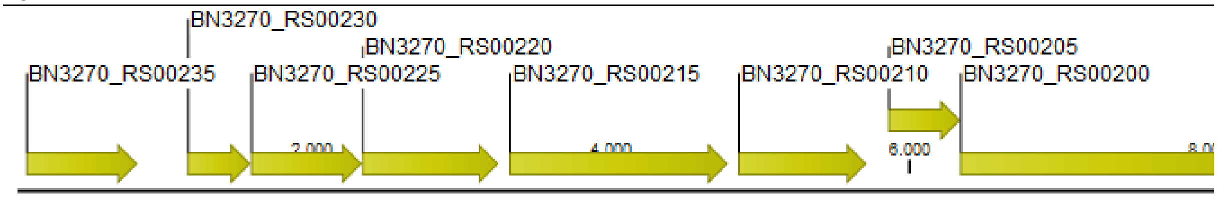

[a], *Eggerthellaceae bacterium* AT8, whole genome shotgun sequence. GenBank: NZ_LN908982.1 (GCF_001486445.1)*. Representation in reverse-complement.

*During preparation of this manuscript this whole genome sequence was removed by RefSeq staff.

Supplemental Table S10: Annotation of the conserved open reading frames (orfs) within the equol biosynthesis gene cluster of ***Lactococcus garvieae* 20-92.**

Locus_tag	Strand	Position[a]	Gene product	Protein ID [sequence length]
**BAJ72746.1 / ORF-US4**	+	46-960	unnamed protein product; ORF-US4	BAJ72746.1 [304 aa]
**l-thdr**	+	1072-2532	tetrahydrodaidzein reductase	BAJ72747.1 [486 aa]
**l-dhdr**	+	2678-3538	dihydrodaidzein reductase	BAJ72748.1 [286 aa]
**BAJ72749.1 / ORF-US1**	+	3635-4138	unnamed protein product; ORF-US1	BAJ72749.1 [167 aa]
**l-dznr**	+	4211-6145	daidzein reductase	BAJ72750.1 [644 aa]
**BAJ72751.1 / ORF-DS1**	+	6279-7658	unnamed protein product; ORF-DS1	BAJ72751.1 [459 aa]
**BAJ72752.1 / ORF-DS2**	+	7888- >9228	unnamed protein product; ORF-DS2 (incomplete)	BAJ72752.1 [447 aa]

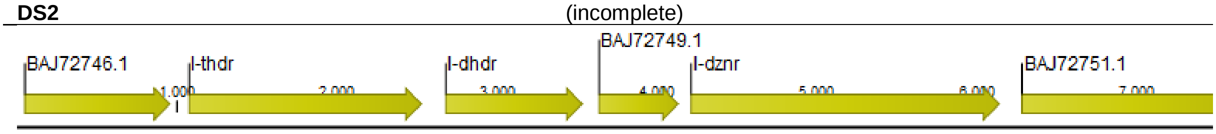

[a], *Lactococcus garvieae ORF-US4*, *l-thdr*, *l-dhdr*, *ORF-US1*, *l-dznr*, *ORF-DS1*, *ORF-DS2* genes, complete and partial cds, strain: 20-92. GenBank: AB593374.

Supplemental Table S11: Annotation of the conserved open reading frames (orfs) within the equol biosynthesis gene cluster of ***Slackia* sp. AUH-JLC159.**

Locus_tag	Strand	Position[a]	Gene product	Protein ID [sequence length]
**dgr**	+	105-2039	daidzein and genistein reductase	AIC80887.1 [644 aa]

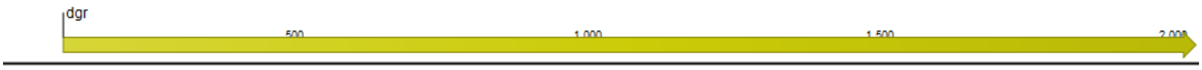

[a], *Slackia* sp. AUH-JLC159 daidzein and genistein reductase (*dgr*) gene, partial cds. GenBank: KJ452760.

Supplemental Table S12: Annotation of the conserved open reading frames (orfs) within the equol biosynthesis gene cluster of ***Slackia* sp. NATTS.**

Locus_tag	Strand	Position[a]	Gene product	Protein ID [sequence length]
**dece-1**	+	490-1950	DHD-to-equol conversion enzyme 1	BAL46928.1 [486 aa]
**dece-2**	+	2059-2907	DHD-to-equol conversion enzyme 2	BAL46929.1 [282 aa]
**ORF**	+	3027-3509	ORF (predicted)	no protein ID [161 aa]
**ddce**	+	3567-5501	daidzein-to-DHD conversion enzyme	BAL46930.1 [644 aa]

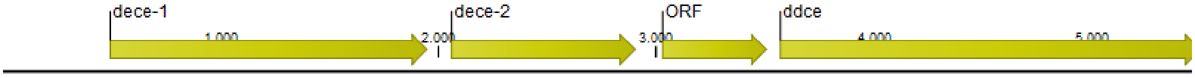

[a], *Slackia* sp. NATTS *dece-1*, *dece-2*, *ddce* genes for DHD to equol conversion enzyme 1, DHD to equol conversion enzyme 2, daidzein to DHD conversion enzyme, complete cds. GenBank: AB646272.1.

Supplemental Data S13: Results of validation (accuracy, intra-day precision, recovery, LOD, LOQ and linearity) of LC-DAD analyses of daidzein, genistein and corresponding microbial metabolites in fermentation samples of pure cultures.
For accuracy and intra-day precision six analyte-free fermentation samples inoculated with *E. lenta* DSM 2243[T] were spiked with a standard mixture of analytes (final concentration in injected samples each 40 µM) and processed as described.
Additionally, six analyte-free fermentation samples inoculated with *E. lenta* DSM 2243[T] and without spiking the standard mixture prior to extraction were processed and spiked during solvation of extraction residue prior to LC-DAD analysis. To calculate recoveries, the peak areas of analytes in these samples were compared to peak areas of analytes in fermentation samples spiked with analytes before extraction. SD, standard deviation.

	**Retention time [min]**	**Accuracy[a] [%]**	**Intra-day precision [%]**	**Recovery (mean ± SD) [%]**	**Limit of detection[b] (LOD) [µM]**	**Limit of quantitation[c] (LOQ) [µM]**
**Daidzein LC-DAD method**						
Daidzein	6.3	103.3	1.4	93.0 ± 1.1	0.06	1.08
Dihydrodaidzein	5.8	102.6	1.4	93.6 ± 1.1	0.06	1.07
Equol	9.1	102.5	1.5	91.9 ± 1.0	0.17	1.09
O-Desmethyl-angolensin	12.1	102.4	1.7	90.2 ± 1.4	0.06	1.11
**Genistein LC-DAD method**						
Genistein	6.4	100.4	1.2	94.5 ± 1.2	0.06	1.06
Dihydrogenistein	5.7	100.9	1.1	95.7 ± 1.1	0.05	1.04
6'-hydroxy-*O*-Desmethyl-angolensin	6.9	99.5	3.5	66.7 ± 2.6	0.08	1.48

[a] corrected for recoveries
[b] calculated for signal/noise = 3 in LC-DAD; values for initial incubation solution and corrected for recoveries
[c] lowest calibration samples (2 µM) in LC-DAD were set as LOQ; values for initial incubation solution and corrected for recoveries

Linearity

To quantify analytes, an external standard calibration was conducted. Therefore, calibration samples (4 levels) in pure solvent were prepared with final concentrations (in injected samples) ranging each from 2.0 to 200 µM. Calibration curves were obtained by linear regression using none weighting for the “Daidzein LC-DAD method” and a weighting of $1/C^2$ for the “Genistein LC-DAD method”. In the validation experiment, the correlation coefficient was used as an indicator for the quality of the calibration curves and was ≥0.999978.
